# Rest and Sleep

**Published:** 1899-10

**Authors:** J. Cam Anderson

**Affiliations:** Halston Bridge, Va.


ARTICLE XIII.

### REST AND SLEEP.

J. CAM ANDERSON, M. D., HALSTON BRIDGE, VA.

This is a fast age as compared with a half or even a quarter of a century ago. Fast, reckless living. In order to keep up in the busy whirl of life, many exert themselves beyon their strength, and many even fall by the wayside, and give up the struggle; many bring on disease and have to drag out a miserable existence, and some few, yes, a great many, as a consequence, "shufflle off this mortal coil" by suicide. There are many routes, and each selects his peculiar way.

Fast living in a fast age. Is there no remedy to help poor, unfortunate victims of the "pride of life?" Yes, largely, there is, if people would only stop and think and apply the remedy. What is it? Brain rest and sleep.

The tendency of the age is more now, than at any other time in the history of this country, to mental overwork and strain, and exhaustion of physical and brain force. "The errors and indiscretions of youth are drafts upon old age, if one should live so long, payable forty years after date, with compound interest." This is a fearful thought. Yet, how true. How true it is, more or less, of every individual.

The circumstances surrounding active professional life, to-day, are such that the nervous system must be of the most perfect character to resist and stand the immense strain made upon it.

When you think of the influences brought to bear upon one in active city business, it is a wonder and a source of surprise that there are not even more cases of insanity from brain exhaustion, leading on to the common fatality, suicide. What will tend to prevent? Brain rest and sleep.

The demand of the times is for a greater amount of brain rest, longer periods from business cares and worries, confusion, excitement, anxiety and responsibilities. We are a nation of dyspeptics, from fast living, over eating, lack of brain rest and sleep.

Working and worrying six days of the week, and oftentimes Sunday, too, all day long, nearly all night, day in and day out. We stand it for awhile, but what is the result? Disease, insanity and death; premature, sad, but true.

It is a scientific fact that one third of our time should be spent in sleep; quiet, restful, natural, refreshing sleep. But, instead of this, how is it? How is it with you, my reader? Think about it and see if you are just to your own self in this particular.

Many men, and especially literary men, writers, preachers, lawyers and doctors, all, more or less, starve the brain incessantly in this matter, until they bring on a partial, and sometimes an incurable insomnia. An irritable man, cross, pettish, peevish, disagreeable and easy "fly off the handle" and get mad as a "wet hen" on the slightest provocation, can nearly always be classed as one who does not get or take sleep enough. A self-composed, placid, quiet, thoughtful, even tempered, genial-natured man, may be known as one who not only insits upon having his full quota of sleep, but one who takes it—eight good, solid hourse of sleep and rest.

From a medical standpoint, dear reader, let me impress upon you that sleep, natural sleep, is the best and only natural tonic to the nervous system, known, and all other so-called tonics, are stimulants. Nature's tonics are exercise, pure air and water, sunshine and sleep, all else are stimulants, and oftentimes harmful, instead of being conducive to health.

Brain rest and sleep, freedom from care, anxiety and responsibility, pure air and water, health exercise, regulated diet, good digestion—and this generally brings it—and full eight hours of sleep, would lengthen the life of the present generation from ten to thirty years.

Go slow and sure, is a good rule, but the age won't let you. Here we go, pell-mell, helter-skelter, on, on, in the mad rush, headlong, heels overhead. What is the result? Sooner or later (and generally sooner), a wreck, body and mind, and probably soul, avarice, love of money. Stop and think. Apply the scripture: "What doth it profit a man if he gain the whole world, and lose his own soul?" or "What shall a man give in exchange for his soul?"

'Tis a good plan for busy brain workers to rest in daytime. Take time to rest and let the brain recuperate. The after-dinner nap, ten or twenty minutes, is good. This is a fact, demonstrated by experience and observation. Rest the body and mind. Give Nature a chance and she will cure you. The physician only aids Nature. When you are sick, so study and give Nature her way and you will get there with both feet and right side up with care. Especially valuable is this momentary resting down on the parlor sofa or out in the wood-shed on the ground, to those whose appetites and digestion must be encouraged, and who are in only a small degree troubled with insomnia, or sleeplessness. Doctor, when you get this class of patients, quiet your patient's nervous system, artificially, naturally, or by imagination, and get sleep, restful sleep. Your patient, when all else is favorable, ten to one, will get well.

### REST AND SLEEP.

The person, or the busy-brain worker who will habitually rest in day time, this is outside of eight hours (good long sixty minutes to the hour) at night, a few minutes, when tired, or worried, will stand his work better and longer, and suffer less, when forced to lose some of the regular hours to sleep. There are some men who have stood terrible strain on the nervous system, and an enormous amount of work, with but little sleep, but all suffered more or less, by it. Some, it is believed, have the power to rest certain portions of the brain, while other parts of brain are in active service. No doubt this is true; but the fact is applicable, however, to a limited few. The vast majority need, and must have an abundance of good old sleep, and the hours spent in sleep in these cases of brain-workers will be more than added to their term of years. As the fellow said, who was slightly intoxicated: "Hurrah, —boys, whooray—hic—I say, hoor—ray, hic—I say—hic hurrah for old Christmas—old Crismus." I say, hurrah for old sleep, good old, healthy, warm sleep; without it we would all soon be dead, and many are slowly dying for the want of it, and solely because, in this fast age, they just won't take the time to take it.

In conclusion, dear reader, let me say, go slow, take time for all things. Take time to think, to eat, to rest, to sleep, and don't rush along in your wild career after this world's goods. Go slow, take your time, "before the silver cord be loosened, or the golden bowl be broken." But be ready when the summons comes, "For ye know not the day nor the hour wherein the Son of man cometh." But work, and work now, and also rest, remembering "the night cometh when no man can work." And at last, when it is ours, in the very nature of things, to lie down and die, let us join in with the immortal Jackson, as he lay dying of wounds in defense of his country, his last memorable words, "Let us pass over the river, and rest under the shade of the trees." Rest, rest, sweet rest.—*Medical Brief.*

